# The Weight of Cardiovascular Diseases: Addressing the Global Cardiovascular Crisis Associated with Obesity

**DOI:** 10.5334/gh.1451

**Published:** 2025-08-21

**Authors:** Francisco Lopez-Jimenez, Mariachiara Di Cesare, Jaynaide Powis, Shreya Shrikhande, Marvellous Adeoye, Elisa Codato, Bin Zhou, Honor Bixby, Natalie Evans, Kyla Lara-Breitinger, Mariana Arellano Rodriguez, Lisa Hadeed, Simon Barquera, Sean Taylor, Pablo Perel, Daniel Pineiro, Jagat Narula, Fausto Pinto

**Affiliations:** 1 Department of Cardiovascular Medicine, Mayo Clinic, Rochester, MN, United States; 2 Institute of Public Health and Wellbeing, University of Essex, Colchester, United Kingdom; 3 World Obesity Federation, London, United Kingdom; 4 World Heart Federation, Geneva, Switzerland; 5 School of Public Health, Imperial College London, London, United Kingdom; 6 Population Health Research Institute, City St George’s, University of London, London, United Kingdom; 7 Center for Nutrition and Health Research, National Institute of Public Health, Cuernavaca, Mexico; 8 Department of Non-Communicable Disease Epidemiology, London School of Hygiene & Tropical Medicine, United Kingdom; 9 Department of Medicine, University of Buenos Aires, Buenos Aires, Argentina; 10 McGovern Medical School, University of Texas Health Sciences Center, Houston, Texas, United States; 11 Santa Maria University Hospital, CAML, CCUL, Faculdade de Medicina da Universidade de Lisboa, Lisbon, Portugal

**Keywords:** Obesity, cardiovascular diseases, body mass index, CVD global data

## Abstract

Obesity is a growing global epidemic with significant implications for cardiovascular diseases (CVD). It couples as an independent risk factor and driver for multiple pathways leading to CVDs. Here we examine obesity’s impact on CVD and propose actionable strategies. Data from the NCD Risk Factor Collaboration (NCD-RisC), Global Burden of Disease (GBD) survey, and regional health surveys databases were used. We examined trends in obesity prevalence and CVD mortality attributable to high body mass index (BMI), disaggregated by sex, geography, socioeconomic status, and urban–rural residence. Evidence from national policy initiatives and clinical management guidelines was also reviewed. As of 2022, over 1 billion people globally were living with obesity. Since 1990 the age-standardised obesity prevalence has doubled among women (from 8.8% to 18.5%) and tripled among men (from 4.8% to 14%). Globally, the number of annual CVD deaths attributable to high BMI (25 kg/m^2^ or over) more than doubled between 1990 and 2021, reaching 1.9 million in 2021. Reducing global obesity to 2019 levels could save an estimated US$2.2 trillion annually by 2060. Positive steps have been made in recent years, with the implementation of several global, national and local initiatives that show promise in tackling obesity and CVDs, in addition to the emergence of potentially game-changing medical interventions, such as glucagon-like peptide-1 receptor agonists (GLP-1RAs). Yet, to tackle obesity and associated CVD, there is a need for a holistic approach across clinical and public health interventions that accounts for the multiple determinants of obesity. We recommend the implementation of evidence-based, cost-effective public health measures, and the incorporation of obesity-specific recommendations into cardiovascular guidelines. Addressing the global cardiovascular crisis linked to obesity will require coordinated efforts from policymakers, healthcare systems, and global health organisations.

## Introduction

Obesity is a global epidemic with significant clinical, economic, and epidemiological consequences (1). Over the past three decades, its prevalence has increased across all regions and income groups, affecting both adults and children. In 2022, almost 900 million adults globally were estimated to be living with obesity, a more than fourfold rise from 1990 (2). In the same year, 159 million children and adolescents had obesity, raising major concerns about early cardiovascular risk (2). Projections indicate that, if the current trajectories persist, nearly two-thirds of adults aged ≥25 years will be classified as overweight or obese by 2050 (3).

**Figure 1:**
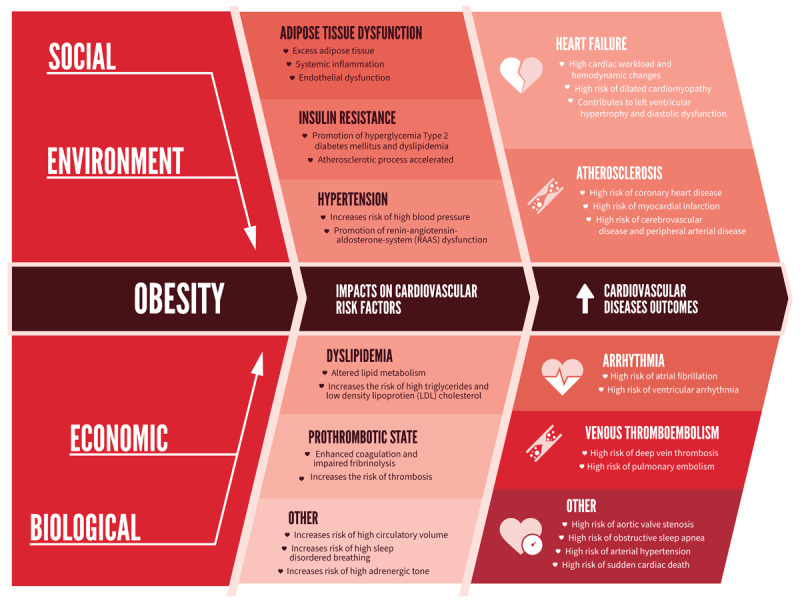
The association between obesity and CVDs.

While recognised as a disease entity, obesity significantly contributes to the development of other non-communicable diseases (NCDs), particularly cardiovascular diseases (CVDs), which constitute the leading cause of global mortality, with almost 20 million deaths a year. Excessive adiposity contributes to cardiometabolic dysregulation through a range of interrelated mechanisms, including insulin resistance, endothelial dysfunction, systemic inflammation, dyslipidemia, and neurohormonal activation leading to CVD complications such as hypertension, heart failure, coronary artery disease, and stroke (Figure 1) (4,5). In 2021, approximately 1.9 million CVD deaths were attributable to high body mass index (BMI), representing nearly 9.8% of all cardiovascular deaths worldwide (6). This proportion has increased in nearly all geographical regions across the world, with low- and middle-income countries (LMICs) facing a disproportionate burden (7).

In 2019, the global economic impact of obesity was estimated to be equivalent to 2.2% of global gross domestic product (GDP), or just under US$2 trillion annually (8), including both direct and indirect costs. Though obesity has economic impacts on all countries, there is a significant variance. In 2019, this ranged from a low of 0.9% of GDP in low-income countries to 2.5% in high-income countries (9). At the regional level, it ranged from 1.1% of GDP in the WHO African Region to 3% in the WHO Region of the Americas in 2019 (8).

If current trends in obesity levels continue, the economic burden is projected to rise to over 3% of GDP per year globally by 2060, with costs expected to triple in high-income countries and increase 23-fold in upper-middle-income countries (8,10).

Despite increasing recognition of the association between obesity and CVD, current responses remain suboptimal. Obesity continues to be frequently misconstrued as a matter of individual lifestyle choice rather than a complicated, multifaceted disorder influenced by biological, social, environmental, and commercial determinants. Furthermore, ongoing stigmatisation and disparities in healthcare impede both preventive and therapeutic initiatives (11,12,13).

The World Heart Federation (WHF), through its global network, is committed to addressing this crisis. Here we summarise the evidence and analysis underlying the World Heart Report 2025 (14). The report analyses global trends in obesity-related CVD, explores sex- and region-specific patterns, identifies key determinants, and evaluates clinical and public health interventions. By doing this, we aim to support cross-sectoral policy action and inform the development of evidence-based strategies for obesity and CVD prevention and management.

## Methods

We relied on epidemiological data from internationally recognised sources, selected based on their methodological robustness, completeness, and availability of disaggregated by sex, age, and geography.

Prevalence estimates for overweight (defined as BMI≥25 kg/m^2^), obesity (defined as BMI≥30 kg/m^2^), and mean body mass index were obtained from the NCD Risk Factor Collaboration (NCD-RisC) (15). Data on cardiovascular mortality and disability-adjusted life years (DALYs) attributable to high BMI were drawn from the Global Burden of Disease (GBD) Study 2021 (16). Additional data, including socioeconomic and demographic indicators, were informed by nationally representative health surveys and policy databases. Different regional definitions (NCD-RisC, WHO, and GBD) were used due to the need to draw from different data sets (Supplementary Figure 1). Data presented are age-standardised to allow for temporal and geographical comparison.

The analytical approach was primarily descriptive. It included assessment of age-standardised prevalence and mortality rates across countries and regions. Data were examined across sex, education level, income group, and urban/rural residence, where available. Temporal trends were evaluated from 1990 to 2021 or 2022 (depending on the source of data used), and visual outputs, such as maps and burden charts, were used to show disparities across regions. See Supplementary Material for full methodological details, including regional definitions and BMI risk thresholds.

## Results

### Global and regional prevalence of obesity

The global prevalence of obesity has more than quadrupled since 1990. In 2022, approximately 878 million adults (over 20-years old) and 159 million children and adolescents were living with obesity worldwide. Between 1990 and 2022, the age-standardised obesity prevalence doubled among women (from 8.8% to 18.5%) and tripled among men (from 4.8% to 14%).

Prevalence varied between countries. In 2022, the highest rates among women were reported in Tonga, American Samoa, and Samoa (75%–81%), and among men in American Samoa, Nauru and Tokelau (67%–70%). The prevalence was also high in many other Pacific and Caribbean Island nations, such as the Bahamas and Saint Kitts and Nevis (38% for men and 55% for women), as well as in the Middle Eastern and North Africa countries like as Qatar and Kuwait (39%–41% for men) and Egypt and Qatar (53%–59% for women).

**Figure 2:**
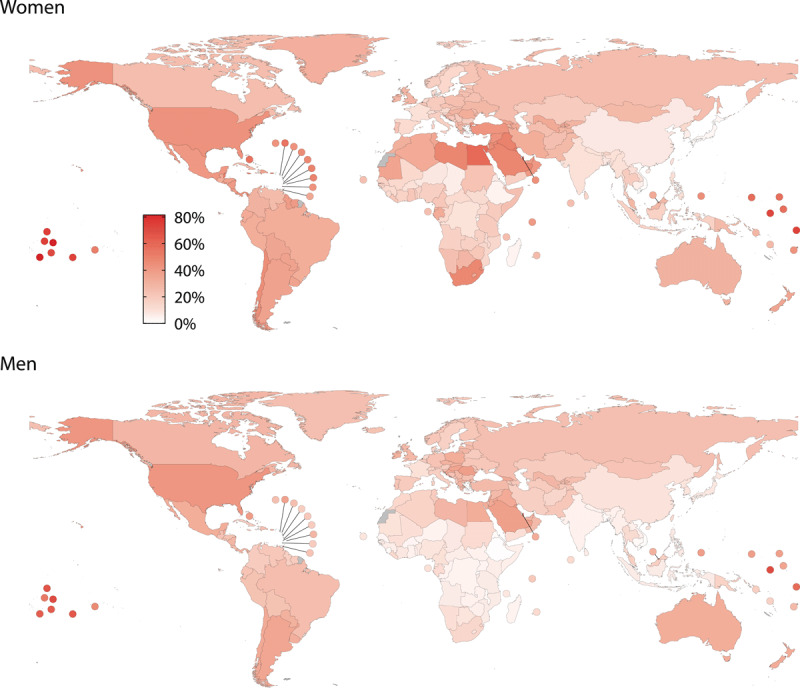
Age-standardised prevalence of obesity in 2022. *Source*: NCD Risk Factor Collaboration.

The lowest rates were observed among women in Vietnam, Timor-Leste, and Japan (2%–4%) and among men in Ethiopia, Timor-Leste and Rwanda (1%–2%). Prevalence also remained below 5% in several other Asian and East African countries (Figure 2).

Obesity increased in nearly every country from 1990 to 2022, with the largest increases observed in the Bahamas for women (from 22.8% to 55.8%) and Romania for men (from 6.7% to 38.3%). Declines in obesity prevalence were rare, occurring only among women in Spain (a 4.6 percentage point decrease), and in France, Moldova, and Lithuania (decreases of 1–2 percentage points).

Childhood obesity demonstrated equally concerning trends. Among school-aged children and adolescents (5–19 years), age-standardised obesity prevalence increased from 1.7% to 6.9% in girls and from 2.1% to 9.3% in boys between 1990 and 2022. This represented 65.1 million girls and 94.2 million boys living with obesity globally in 2022.

### Cardiovascular mortality attributable to high BMI

Globally, the number of annual CVD deaths attributable to high BMI (at least 25 kg/m^2^ or over) more than doubled between 1900 and 2021, reaching 1.9 million in 2021, more than half of the total 3.7 million deaths from high BMI and 9.8% of total CVD deaths.

Globally, age-standardised CVD mortality attributable to high BMI was 22.8 deaths per 100,000 people in 2021, moderately lower than in 1990 due to the decline in overall CVD mortality.

In 2021, the global age-standardised CVD mortality rate attributable to high BMI was 22.8 deaths per 100,000 population, a moderate decline from 1990, reflecting reductions in overall CVD mortality. However, large regional disparities remain. The highest age-standardised CVD mortality rates attributable to high BMI were observed in North Africa and the Middle East (67.5 per 100,000), while the lowest were in high-income countries (14.4 per 100,000) (see Supplementary Figure 2).

There were an estimated 300,000 more deaths globally attributable to high BMI among women compared with men. High BMI is the eighth most important risk factor for deaths from CVD globally. Since 1990, its rank has increased in every region except South Asia and Southeast Asia, East Asia, and Oceania, where it remained unchanged.

At the national level, high BMI was the third leading CVD risk factor in 14 countries and ranked fourth in an additional 44 countries. Among women, 10.8% of CVD deaths were attributable to high BMI compared to 8.9% in men. A similar sex difference was observed for CVD disability-adjusted life years (DALYs), with high BMI contributing 11.6% of CVD DALYs in women and 9.8% in men. Globally, high BMI ranked as the fifth leading contributor to CVD DALYs for women, and seventh for men.

### Sex differences

Globally, the age-standardised prevalence of obesity in 2022 was higher in women than in men by 4.5 percentage points, a figure that has remained largely stable since 1990. Regionally, the obesity disparity between women and men was largest in Central Asia, the Middle East, and North Africa, where 40% of women live with obesity compared with 25% of men. Across all regions except Europe, obesity prevalence was higher among women than men.

Obesity prevalence was also higher in women than men in South Asia, Southeast Asia, Latin America and the Caribbean, Oceania, and sub-Saharan Africa. In Central and Eastern Europe, the high-income western region and East Asia and the Pacific, obesity prevalence was similar between women and men.

**Figure 3:**
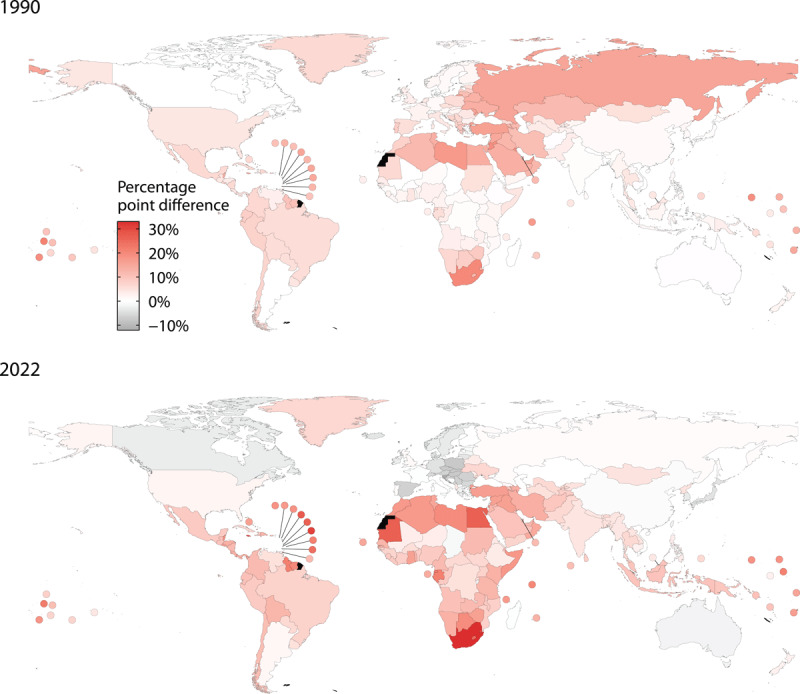
Absolute difference in the age-standardised prevalence of obesity between women and men in 1990 and 2022. Note: A positive difference indicates the prevalence is higher among women; a negative difference indicates the prevalence is higher among men. *Source*: NCD Risk Factor Collaboration.

In 2022, women had a higher prevalence of obesity than men in over three-quarters of countries worldwide (Figure 3), with the difference exceeding 30 percentage points in South Africa, Jamaica and Saint Vincent, and the Grenadines. From 1990 to 2022, the female–male gap in obesity grew larger in most countries in sub-Saharan Africa, Latin America and the Caribbean, Southeast Asia, and in some countries in Central Asia, the Middle East, and North Africa. In contrast, the female–male gap in obesity shrank in all countries in Central and Eastern Europe, East Asia, the Pacific, and most high-income western countries. Males have a higher obesity prevalence in 39 countries worldwide.

### Urban–rural patterns

The global rise in age-standardised mean BMI from 1985 to 2017 was largely driven by increases in rural populations, challenging conventional assumptions about urbanisation and obesity. While urban residents in many low- and middle-income regions maintained higher mean BMI than rural counterparts, faster increases in rural areas resulted in rural-to-urban BMI convergence in most regions.

In South Asia, the urban–rural BMI gap decreased from 3.2 kg/m^2^ for women and 3.0 kg/m^2^ for men in 1985 to 1.9 kg/m^2^ and 1.2 kg/m^2^, respectively, by 2017. Similar convergence patterns were observed in Latin America and the Caribbean, East and Southeast Asia, Central Asia, the Middle East, and North Africa for women, and Oceania for men.

Sub-Saharan Africa showed a divergent pattern, with urban–rural BMI gaps widening among women from 2.6 kg/m^2^ in 1985 to 3.2 kg/m^2^ in 2017. Some countries in this region, including Niger and Burkina Faso, had larger urban–rural BMI gaps than any other countries globally. In high-income countries, rural areas have persistently showed higher BMI than urban areas since 1985, reflecting rural economic and social disadvantage (Supplementary Figure 3).

### Education

**Figure 4:**
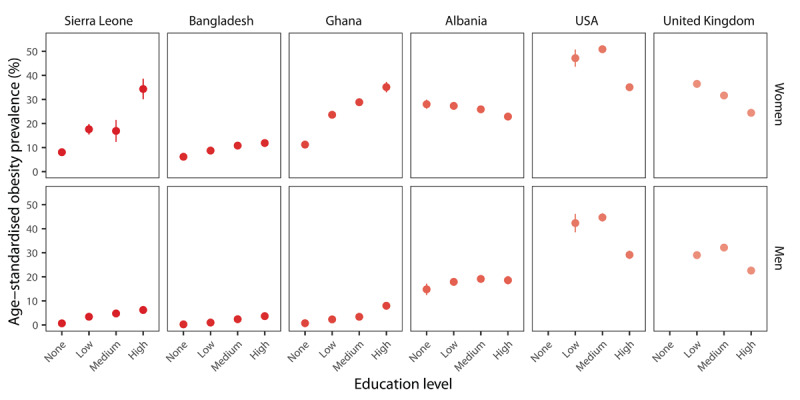
Age-standardised prevalence of obesity by education level in adults aged over 25 years in select countries. *Note*: Country income groupings for this figure are as follows – Low-income: Sierra Leone; Lower-middle-income: Bangladesh and Ghana; Upper-middle-income: Albania; High-income: USA and the United Kingdom. Data sources: All data are based on publicly available nationally representative health examination surveys, including – Sierra Leone, DHS 2019; Bangladesh, DHS 2022; Ghana, DHS 2022; Albania, DHS 2017–2018; United Kingdom, Health Survey for England 2017–2019, USA NHANES, 2021–2023.

Education levels are strongly associated with obesity prevalence, though the direction and strength of this relationship vary by country income level. For example, in higher-income countries, the prevalence of obesity is higher among people with lower levels of education, whereas in low-income and lower-middle-income countries, the prevalence of obesity is higher among people with higher levels of education (Figure 4).

### Clinical management

Clinicians play a pivotal role in preventing and managing CVD by addressing obesity, identifying individualised, evidence-based strategies to promote weight loss and mitigate CVD risk, including through referral to specialist clinicians (17).

Structured lifestyle interventions remain the first-line clinical approach (18,19). Multicomponent programmes involving dietary modification, increased physical activity, and behavioural support are considered the most effective non-pharmacological strategy (19).

Medications for obesity, when used in conjunction with lifestyle interventions, can be effective in increasing weight loss and, when used appropriately, maintaining it in the long term (20). These medications can include stimulants, central nervous system acting agents, and gastrointestinal malabsorption agents. Additionally, these medications have demonstrated effectiveness (5%–10% weight loss) but may carry potential side effects, which can be harmful for individuals with cardiovascular conditions (21).

Recently, more novel treatments known as glucagon-like peptide-1 receptor agonists (GLP-1RAs) and their analogues (originally developed to treat diabetes) are increasingly used in treatment plans (22,23,24). GLP-1RAs are naturally occurring incretins released by the intestine after a meal that work by delaying gastric emptying, thus acting peripherally to decrease hunger and increase insulin sensitivity, among other effects (25). GLP-1RAs and their analogues are shown to treat cardiometabolic disease by improving outcomes across several areas including weight loss, waist circumference, glucose control, dyslipidemia, diabetes, blood pressure, and inflammation decreasing major adverse cardiovascular events for secondary prevention (24), and improving symptoms in patients with heart failure with preserved ejection fraction (HFpEF) (26,27). There is growing evidence that these classes of medications benefit all organ systems through decreasing inflammation and increasing weight loss (28). A recent meta-analysis of people treated with GLP-1RAs compared to a placebo reported the reduction in various cardiovascular outcomes in people with type 2 diabetes (67,769 participants across 10 trials), and in people with established CVD and no type 2 diabetes (17,604 from one trial). Among people with type 2 diabetes who took GLP-1RAs a 14% reduction in major adverse cardiovascular events (MACE) and cardiovascular death, and a 13% reduction in hospitalisation for heart failure were found. In people with CVD and no type 2 diabetes, GLP-1RAs reduced major adverse cardiovascular events (MACE) and non-fatal myocardial infarction by 20% and 28%, respectively (28).

Alternatively, surgical interventions, such as bariatric surgery, represent an effective long-term therapeutic approach for obesity, with approximately 90% of patients experiencing a weight reduction of 15%–40% and a concomitant improvement in obesity-related comorbidities (29,30).

### Public health interventions

Public health interventions, whether community-focussed or system-level, play a vital role in reducing the incidence and impact of obesity and CVD (31). Government-led initiatives targeting the food environment, marketing practices, taxation, and health promotion have demonstrated measurable impacts and serve as examples for wider adoption. In the Netherlands, for example, the city of Amsterdam implemented the Healthy Weight approach, a multisectoral programme involving public health, urban planning, sport, education, welfare, and economic sectors (32). Within 10 years of implementation, evaluations show reduced childhood obesity rates from 21% to 18.7% (32). Mexico introduced a sugar-sweetened beverage tax in 2014, applying a rate of one Mexican peso per litre, approximately 10% of the price of sugary drinks (33). This fiscal measure aimed to reduce consumption of high-sugar products (34).

In Japan, the nationally mandated Shokuiku food education programme was introduced in 2005 (35). It includes classroom-based nutrition education, balanced school meals, and student involvement in food preparation and planning. The intervention targets the development of lifelong healthy eating behaviours through early and sustained engagement.

In Brazil, front-of-pack nutrition labelling regulations approved in 2020 require clear and simple icons to indicate high levels of saturated fat, added sugar, or sodium (36). This labelling system is designed to enable consumers to make informed dietary choices and reduce the consumption of ultra-processed foods (37).

The Healthy Food Financing Initiative (HFFI) in the United States provides funding to eligible organisations that are planning to develop a food retail outlet or food supply chain that will improve access to staple and perishable foods in underserved areas (38).

In the United Kingdom, Gateshead Council introduced a planning policy in 2015 to restrict the opening of new fast-food outlets (39). The objective was to reduce childhood obesity from 22.6% in 2015 to below 10% by 2025 (40).

The ‘Eat Right India’ campaign, launched in 2018 by the Food Safety and Standards Authority of India (FSSAI), designed to promote healthy eating and raise awareness about the importance of balanced diets to prevent obesity (41). It uses mass media, including TV, radio and digital platforms, to spread messages on nutrition, food safety, and the dangers of overeating (42).

Finland’s national school meal programme guarantees free balanced meals to all students in pre-primary, primary, lower secondary, and upper secondary education (43). The policy supports equitable access to healthy food for children and adolescents and reinforces dietary education through daily practice.

## Discussion

This manuscript presents a comprehensive analysis of the World Heart Report 2025 on the relationship between obesity and cardiovascular diseases, revealing the magnitude of what has become one of the most pressing global health crises of our time. The analysis reveals a marked increase in obesity prevalence across all age groups and regions, significantly contributing to CVD morbidity and mortality, economic strain, and health inequities, particularly in LMICs. There is an urgent need for integrated, multisectoral strategies to address this multifaceted disease, moving beyond simplistic views of obesity as a lifestyle issue. By synthesising global trends, disparities, and actionable insights, this report offers a roadmap for stakeholders to mitigate obesity’s impact on cardiovascular health.

The global burden of CVD attributable to high BMI has increased steadily, now ranking among the top 10 contributors to CVD mortality and DALYs. It reveals the fundamental inadequacy of treatment strategies that have not addressed the metabolic and behavioural risk factors fueling CVD epidemics. Obesity is not only a prominent and independent risk factor for a range of cardiovascular outcomes but also acts as a mediator of other CVD risks, particularly hypertension, dyslipidemia, and type 2 diabetes.

We highlighted the persistent and often widening inequality in obesity burden across demographic and geographic subgroups. These inequalities are not incidental but structural, tied to broader systems of food production and distribution, labour markets, health infrastructure, and cultural norms. In many LMICs, for example, women face higher exposure to obesogenic environments due to limited access to safe spaces for physical activity, cultural pressures around body image, and economic constraints that affect dietary choices (44).

Perhaps most concerning is the dramatic rise in childhood obesity, with prevalence increasing from 1.7% to 6.9% in girls and 2.1% to 9.3% in boys between 1990 and 2022 (2). This represents not just a current health crisis but a demographic time bomb that will drive a dramatic increase in adult CVD burden over the coming decades. Children with obesity face a 40% higher risk of CVD in midlife, and when combined with other risk factors, this risk can increase two- to nine-fold (45).

We emphasised the role of the social determinants of health, for example, income and education, as critical factors contributing to the epidemic of obesity and the associated burden of cardiovascular diseases. Differential access to healthy and nutritious foods, time and space for physical activity, access to healthcare services, and exposure to chronic stress explain the highest prevalences of obesity observed among low socio-economic and marginalised groups in high income settings. While social determinants have received considerable attention, commercial determinants represent a critical yet under-addressed driver of the global obesity epidemic (46,47). The food industry’s aggressive marketing of ultra-processed foods and sugary beverages, particularly when targeted at children, contributes significantly to non-communicable disease mortality worldwide. This commercial influence operates through sophisticated marketing strategies that shape food preferences and consumption patterns from an early age. Adding another layer of complexity, biological determinants, including genetic predispositions and alterations in gut microbiota, suggest promising avenues for personalised medicine approaches to obesity management (48).

The implications of these findings are far-reaching, particularly for LMICs, where health systems face the dual challenge of obesity and undernutrition, often termed the ‘double burden of malnutrition’. This convergence exacerbates resource constraints, as LMIC health systems allocate limited budgets to both infectious and NCDs. The economic strain, driven by healthcare costs and productivity losses, threatens sustainable development, undermining progress towards sustainable development goals (SDGs), notably SDG 3 (good health and well-being) and SDG 1 (no poverty). For cardiologists, these trends necessitate integrating obesity management into routine CVD care through screening, counselling, and multidisciplinary approaches. Public health systems must prioritise prevention, addressing upstream determinants to reduce the downstream CVD burden.

Interventions to address obesity and its CVD consequences cut across clinical and public health domains. Clinically, GLP-1RAs have shown promise in reducing body weight and CVD risk, while bariatric surgery, effective for severe obesity, is widely used in HICs but remains inaccessible in LMICs due to cost and capacity constraints.

The implementation of public health interventions targeting obesity at the national and local levels has shown effectiveness in multiple contexts and provides valuable insights for global application. Implementation of multisectoral strategies encompassing, for example, urban planning, food environment improvements, and educational campaigns, has resulted in decreased prevalence of obesity among children (49,50,51). On the other side, fiscal measures, such as taxes on unhealthy products (e.g., sugar-sweetened beverages), have been successful through product reformulation and consumer behaviour change.

These national and local efforts reflect growing recognition of obesity as a global health priority, yet the scale and consistency of action remain inadequate. Global frameworks such as the WHO Acceleration Plan to Stop Obesity (31) and the Global Action Plan for the prevention and control of noncommunicable diseases (52) provide structured roadmaps and technical guidance for governments. If coordinated and evidence-based interventions are scaled globally, the potential economic impact of reversing obesity trends is substantial. Estimates indicate that reducing overweight and obesity prevalence to 2019 levels could yield annual global savings of US$2.2 trillion in healthcare and productivity-related costs between 2020 and 2060 (8).

Finally, we acknowledge that reliance on BMI as a primary metric, while practical for global surveillance, overlooks visceral fat distribution and muscle mass, potentially underestimating CVD risk in populations like South Asians, who develop complications at lower BMI thresholds (53). The 2025 Lancet Commission on obesity (54) recommends incorporating direct measures of adiposity, such as waist circumference or body fat percentage, alongside BMI to improve diagnostic accuracy. The Commission proposes a novel diagnostic framework categorising obesity into pre-clinical and clinical obesity based on the presence or absence of health conditions linked to excessive body fat. This approach aims to better identify individuals at risk for obesity-related complications and inform more targeted interventions, potentially improving the cost-effectiveness of obesity treatments and prioritizing treatment based on the burden of obesity-associated conditions.

In conclusion, obesity prevalence in the world has increased significantly in recent decades and contributes to the global burden of CVD. Addressing the global cardiovascular crisis linked to obesity will require coordinated efforts from policymakers, healthcare systems, and global health organisations. The World Heart Federation calls for all countries and stakeholders to urgently work together to accelerate efforts to reduce the mortality and morbidity burden of obesity and CVDs through the implementation of evidence-informed public health and clinical interventions. The World Heart Federation recommendations include:

Public health interventions

1. All countries should implement evidence-based, cost-effective public health measures to address obesity and CVD, and prioritise comprehensive, integrated and equity-focussed policies guided by national data on how determinants vary across population groups. Implementation should draw on global frameworks and tools, including WHO’s ‘Acceleration Plan to Stop Obesity’, the ‘Global Action Plan for the prevention and control of noncommunicable diseases’, and ‘The Global Strategy on Diet, Physical Activity and Health’. Priority actions include national obesity roadmaps with defined targets, multisectoral policies addressing the broad scope of obesity determinants, and ensuring greater integration of obesity prevention and treatment into primary healthcare services.
2. Public health campaigns and policies must counteract the stigmatisation of obesity, including through the use of person-first language, recognising that stigma is a hindrance to tackling obesity and CVD. This includes acknowledging the broad determinants of obesity and implementing legislation to address discrimination against people with obesity and overweight. To achieve best results, the design of campaigns and policy initiatives should involve people living with obesity and CVD.

Clinical interventions

3. Cardiovascular guidelines should incorporate obesity-specific recommendations to ensure CVD management is adequately adapted for people living with obesity. This includes equipping health professionals with the knowledge, guidance, and tools necessary to optimally prevent, manage, and treat obesity.
4. Health professional education and health system strengthening initiatives for obesity and CVD should focus on promoting person-centred, integrated care, and where possible be responsive to disparities in obesity risk, prevalence, and access to care.
5. Governments should increase efforts to expand the availability and affordability of obesity medications, such as GLP-1RAs, including through:
  - Ensuring they are covered under public health insurance plans to make them affordable and available for a broader population of persons with obesity and related conditions like type 2 diabetes, in alignment with clinical practice guidelines.
  - Undertaking cost-reduction initiatives via negotiation with pharmaceutical companies to reduce the cost of GLP-1RAs or incentivising generic alternatives once patents expire. Governments can also explore bulk purchasing options to reduce drug prices for citizens.
6. Governments should increase efforts to expand the public awareness, availability and accessibility of specialised lifestyle modification programmes, including those targeting children and adolescents. Examples of these specialised programmes include nutritional, physical activity, weight loss, and cardiac rehabilitation programmes.

## Additional File

The additional file for this article can be found as follows:

10.5334/gh.1451.s1 Supplementary File. Supplementary Material.
