# Supplementary material for: The Weight of Cardiovascular Diseases: Addressing the Global Cardiovascular Crisis Associated with Obesity
Source: Glob Heart. 2025 Aug 21;20(1):68. doi: 10.5334/gh.1451 (PMC12372701; doi:10.5334/gh.1451)

Supplementary Material

Supplementary Material...1
1. Data sources 2
2. Regional classification 2
Figure 1 - World Health Organisation regional classification 3
Table 1 - GBD regional classification...4
Table 2- NCD-RisC regional classification..6
3. Figures...7
Figure 2 - World Health Organization recommendations for body mass index and waist circumference cut-off points for overweight or obesity, and association with disease risk 7
Figure 3 - Age-standardised CVD mortality attributable to high-BMI by income level, both sexes combined 8
Figure 4 - Regional trends in age-standardised mean BMI from 1985 to 2017, by urban and rural place of residence...10

1. Data sources

Data sources were selected according to some key criteria. Primarily, sources needed to:
a. Be well-utilized and reputable.
b. Be based on robust methodology.
c. Provide data according to sex, and country and/or region,
urban/rural.
Among sources that met these criteria, those with the most complete and rich data (e.g. fewest gaps) were selected. In all cases, the most recent data available from the selected source was used. Data on prevalence of overweight and obesity and mean BMI are from NCD Risk Factor Collaboration (NCD-RisC). Data on attributable mortality due to high-BMI are from the Global Burden of Disease (GBD).

2. Regional classification

Several different ways of grouping countries into regions exist. Three different classifications for global regions are used at different points in the paper due to the need to draw from different data sets. These are noted where relevant—either in the text or figures—and include regional classification from the World Health Organization (WHO), the Global Burden of Disease , and from the NCD Risk Factor Collaboration (NCD-RisC).

Appendix Figure 1 - World Health Organisation regional classification

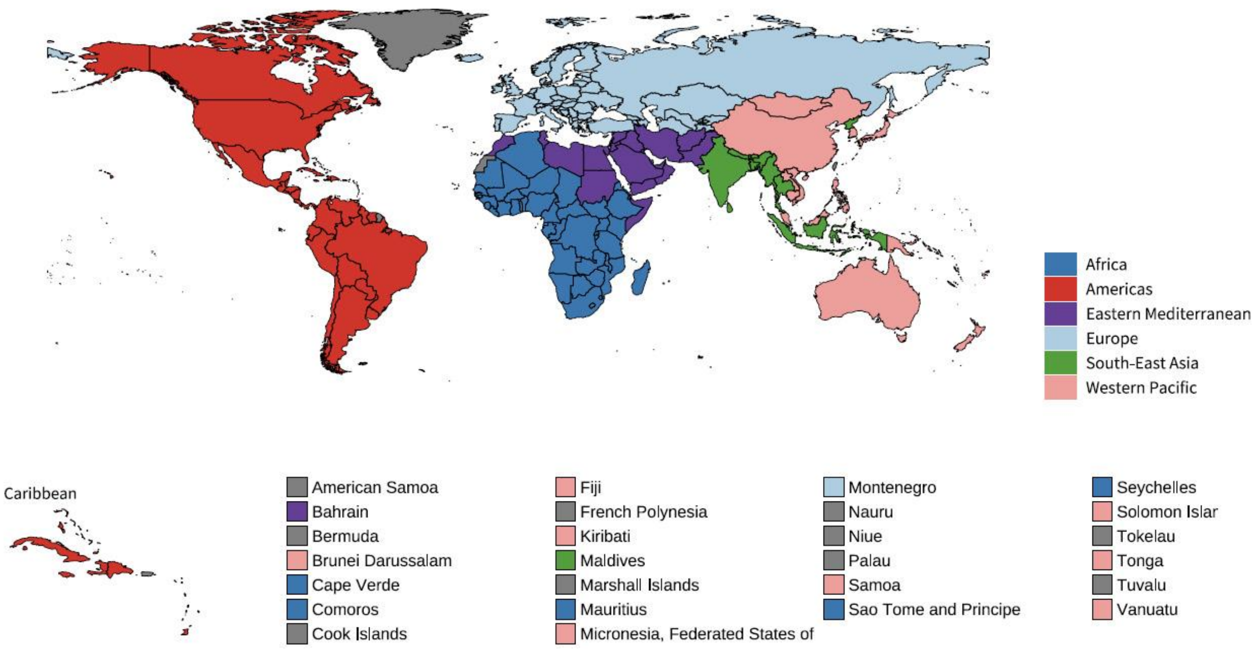

Appendix Table 1 - GBD regional classification

SUPER-REGION	REGION (COUNTRIES)
**CENTRAL EUROPE, EASTERN EUROPE, AND CENTRAL ASIA**	**Central Asia:** Armenia, Azerbaijan, Georgia, Kazakhstan, Kyrgyzstan, Mongolia, Tajikistan, Turkmenistan, Uzbekistan<br>**Central Europe:** Albania, Bosnia and Herzegovina, Bulgaria, Croatia, Czechia, Hungary, Montenegro, North Macedonia, Poland, Romania, Serbia, Slovakia, Slovenia, Belarus, Estonia, Latvia, Lithuania, Republic of Moldova, Russian Federation, Ukraine
**HIGH-INCOME**	**Australasia:** Australia, New Zealand<br>**High-Income Asia Pacific:** Brunei Darussalam, Japan, Republic of Korea, Singapore<br>**High-Income North America:** Canada, United States of America<br>**Southern Latin America:** Argentina, Chile, Uruguay<br>**Western Europe:** Andorra, Austria, Belgium, Cyprus, Denmark, Finland, France, Germany, Greece, Greenland, Iceland, Ireland, Israel, Italy, Luxembourg, Malta, Monaco, Netherlands, Norway, Portugal, San Marino, Spain, Sweden, Switzerland, United Kingdom
**LATIN AMERICA AND THE CARIBBEAN**	**Andean Latin America:** Bolivia, Ecuador, Peru<br>**The Caribbean:** Antigua and Barbuda, Bahamas, Barbados, Belize, Bermuda, Cuba, Dominica, Dominican Republic, Grenada, Guyana, Haiti, Jamaica, Puerto Rico, Saint Kitts and Nevis, Saint Lucia, Saint Vincent and the Grenadines, Suriname, Trinidad and Tobago, United States Virgin Islands<br>**Central Latin America:** Colombia, Costa Rica, El Salvador, Guatemala, Honduras, Mexico, Nicaragua, Panama, Venezuela<br>**Tropical Latin America:** Brazil, Paraguay
**SOUTH ASIA**	**South Asia:** Bangladesh, Bhutan, India, Nepal, Pakistan
**NORTH AFRICA AND MIDDLE EAST**	**North Africa and Middle East:** Afghanistan, Algeria, Bahrain, Egypt, Iran (Islamic Republic of), Iraq, Jordan, Kuwait, Lebanon, Libya, Morocco, Oman, Palestine, Qatar, Saudi Arabia, Sudan, Syrian Arab Republic, Tunisia, Turkey, United Arab Emirates, Yemen
**SOUTHEAST ASIA, EAST**	**East Asia:** China, Democratic People's Republic of Korea, Taiwan (Province of China)

**ASIA, AND OCEANIA**	**Oceania:** American Samoa, Cook Islands, Fiji, French Polynesia, Guam, Kiribati, Marshall Islands, Micronesia (Federated States of), Nauru, Niue, Northern Mariana Islands, Palau, Papua New Guinea, Samoa, Solomon Islands, Tokelau, Tonga, Tuvalu, Vanuatu<br>**Southeast Asia:** Cambodia, Indonesia, Lao PDR, Malaysia, Maldives, Mauritius, Myanmar, Philippines, Seychelles, Sri Lanka, Thailand, Timor-Leste, Vietnam
**SUB-SAHARAN AFRICA**	**Central Sub-Saharan Africa:** Angola, Central African Republic, Congo, Democratic Republic of the Congo, Equatorial Guinea, Gabon<br>**Eastern Sub-Saharan Africa:** Burundi, Comoros, Djibouti, Eritrea, Ethiopia, Kenya, Madagascar, Malawi, Mozambique, Rwanda, Somalia, South Sudan, Uganda, United Republic of Tanzania, Zambia<br>**Western Sub-Saharan Africa:** Benin, Burkina Faso, Cote d'Ivoire, Cabo Verde, Cameroon, Chad, Gambia, Ghana, Guinea, Guinea-Bissau, Liberia, Mali, Mauritania, Niger, Nigeria, Sao Tome and Principe, Senegal, Sierra Leone, Togo<br>Southern Sub-Saharan Africa: Botswana, Eswatini, Lesotho, Namibia, South Africa, Zimbabwe

Appendix Table 2- NCD-RisC regional classification

SUPER-REGION	REGION (COUNTRIES)
**CENTRAL AND EASTERN EUROPE**	**Central Europe:** Albania, Bosnia and Herzegovina, Bulgaria, Croatia, Czechia, Hungary, Montenegro, North Macedonia, Poland, Romania, Serbia, Slovakia, Slovenia<br>**Eastern Europe:** Belarus, Estonia, Latvia, Lithuania, Moldova, Russian Federation, Ukraine
**CENTRAL ASIA, MIDDLE EAST AND NORTH AFRICA**	**Central Asia:** Armenia, Azerbaijan, Georgia, Kazakhstan, Kyrgyzstan, Mongolia, Tajikistan, Turkmenistan, Uzbekistan<br>**Middle East and North Africa:** Algeria, Bahrain, Egypt, Iran, Iraq, Jordan, Kuwait, Lebanon, Libya, Morocco, Oman, Qatar, Saudi Arabia, State of Palestine, Syrian Arab Republic, Tunisia, Türkiye, United Arab Emirates, Yemen
**HIGH-INCOME WESTERN**	**High-income English-speaking countries**: Australia, Canada, Ireland, New Zealand, United Kingdom, United States of America<br>**Northwestern Europe:** Austria, Belgium, Denmark, Finland, Germany, Greenland, Iceland, Luxembourg, Netherlands, Norway, Sweden, Switzerland<br>**Southwestern Europe:** Andorra, Cyprus, France, Greece, Israel, Italy, Malta, Portugal, Spain
**LATIN AMERICA AND THE CARIBBEAN**	**Andean Latin America:** Bolivia, Ecuador, Peru<br>The Caribbean: Antigua and Barbuda, Bahamas, Barbados, Belize, Bermuda, Cuba, Dominica, Dominican Republic, Grenada, Guyana, Haiti, Jamaica, Puerto Rico, Saint Kitts and Nevis, Saint Lucia, Saint Vincent and the Grenadines, Suriname, Trinidad and Tobago<br>**Central Latin America:** Colombia, Costa Rica, El Salvador, Guatemala, Honduras, Mexico, Nicaragua, Panama, Venezuela<br>**Southern Latin America:** Argentina, Brazil, Chile, Paraguay, Uruguay
**OCEANIA**	**Melanesia:** Fiji, Papua New Guinea, Solomon Islands, Vanuatu<br>**Polynesia and Micronesia:** American Samoa, Cook Islands, French Polynesia, Kiribati, Marshall Islands, Micronesia, Nauru, Niue, Palau, Samoa, Tokelau, Tonga, Tuvalu
**SOUTH ASIA**	**South Asia:** Afghanistan, Bangladesh, Bhutan, India, Nepal,

	Pakistan, Sri Lanka
**EAST AND SOUTHEAST ASIA AND THE PACIFIC**	**East Asia and the Pacific:** China, Japan, Singapore, South Korea, Taiwan<br>**Southeast Asia:** Brunei Darussalam, Cambodia, Indonesia, Lao PDR, Malaysia, Maldives, Myanmar, North Korea, Philippines, Thailand, Timor-Leste, Vietnam
**SUB-SAHARAN AFRICA**	**Central and southern Africa:** Angola, Botswana, Central African Republic, Congo, DR Congo, Equatorial Guinea, Gabon, Namibia<br>**East Africa:** Burundi, Comoros, Djibouti, Eritrea, Eswatini, Ethiopia, Kenya, Lesotho, Madagascar, Malawi, Mozambique, Rwanda, Somalia, South Sudan, Sudan, Tanzania, Uganda, Zambia, Zimbabwe<br>**West Africa:** Benin, Burkina Faso, Cabo Verde, Cameroon, Chad, Côte d’Ivoire, Gambia, Ghana, Guinea, Guinea Bissau, Liberia, Mali, Mauritania, Niger, Nigeria, Sao Tome and Principe, Senegal, Sierra Leone, Togo<br>**Other sub-Saharan Africa:** Mauritius, Seychelles, South Africa

3. Figures and Table

Appendix Table 2 - World Health Organization recommendations for body mass index and waist circumference cut-off points for overweight or obesity, and association with disease risk

**Classification**	**Body mass index (kg/m^2)**	**Disease risk (relative to normal weight and waist circumference)**	
**Caucasian populations**		Men <102 cm	Men >102 cm
		Women <88 cm	Women >88 cm
Underweight	<18.5	Very high	—
Healthy weight	18.5–24.9	—	High
Overweight	25.0–29.9	Increased	High
Obesity class I	30.0–34.9	High	Very high
Obesity class II (morbid obesity)	35.0–39.9	Very high	Very high
Obesity class III (severe obesity)	≥40.0	Extremely high	Extremely high
**South Asian, Chinese and Japanese populations**		Men <90 cm	Men >90 cm
		Women <80 cm	Women >80 cm
Underweight	<18.5	Low (increased risk of other clinical problems)	Average
Healthy weight	18.5–22.9	Average	Increased
Overweight (at risk)	23.0–24.9	Increased	Moderate
Obesity class I	25.0–29.9	Moderate	Severe
Obesity class II	≥30.0	Severe	Very Severe

Source: https://academic.oup.com/eurjpc/article/29/17/2218/6675714

Appendix Figure 3 - Age-standardised CVD mortality attributable to high-BMI by income level, both sexes combined

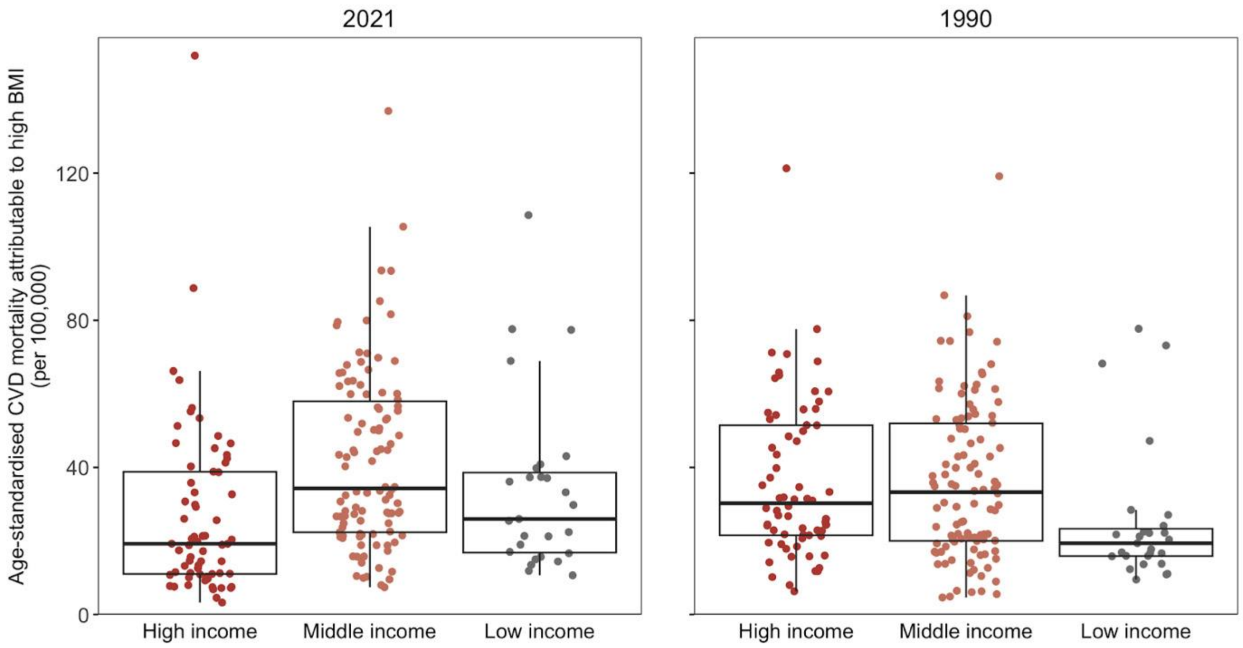

Appendix Figure 4 - Regional trends in age-standardised mean BMI from 1985 to 2017, by urban and rural place of residence

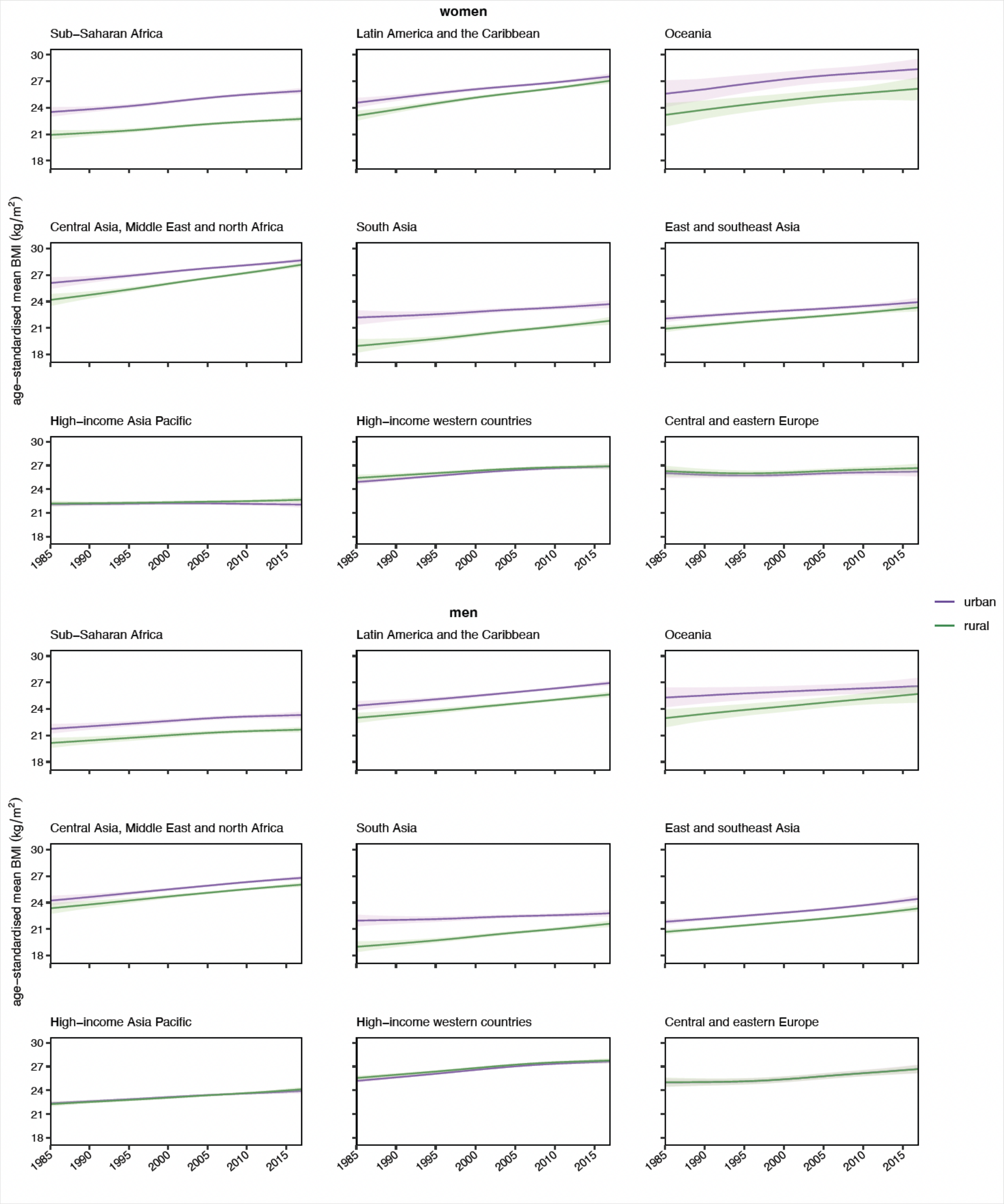

Supplement: Supplementary File. — Supplementary Material. [file gh-20-1-1451-s1.pdf]
